# Numerical calculation finds the revised elastic field of an edge dislocation to be incorrect

**DOI:** 10.1073/pnas.2517060122

**Published:** 2025-12-03

**Authors:** Dallas R. Trinkle

**Affiliations:** ^a^Department of Materials Science and Engineering, University of Illinois at Urbana-Champaign, Urbana, IL 61801

The recent paper (1) claims that there are three issues with the classic linear elastic solution for an edge dislocation, and proposes solutions to these problems. First, the authors posit that the *u*_*y*_ displacement cannot have a logarithmic dependence on distance as there are same direction displacements above and below the slip plane; they add a line force that cancels the logarithmic displacements. However, adding a line force will produce a nonequilibrium solution. Second, they discuss constant displacements that shift the origin, and finally the authors show that if the reference crystal positions are used to compute the displacements, the dislocation lacks crystalline symmetry. They propose adding a +b/2 dislocation cut above and a −b/2 dislocation cut below. An alternate solution (2) showed that a single cut along the −*x* direction with displacements computed using the dislocation positions is symmetric; this requires that the atomic positions be computed self-consistently. Using dislocation positions also removes the origin shift.

The new elastic solution with the line force and no logarithmic dependence on distance can be tested numerically against a large, relaxed atomistic computation of a dislocation. Tungsten is elastically isotropic, and an EAM potential (3) allows efficient computation. Using approximately 1.4 million atoms, two initial $a_{0}\left[100\right]\left(010\right)$ edge dislocation geometries were created: one with the classic (TLE) solution and another with the new solution. The cylindrical simulation geometries have a radius of 1,510 Å; atoms out to 1,500 Å relax, while the outer “skin” of 10 Å is clamped at the initial elastic solution positions. A Hessian-free truncated Newton method in LAMMPS (4) relaxes atoms until all forces are below $10^{-8}$ eV/Å.

The stress fields and line energies of the relaxed dislocations are compared with the two elastic solutions. The TLE solution summed squared displacement during relaxation is $0.479\;b^{2}$, while the new solution is $1,152\;b^{2}$; this shows a significant amount of relaxation for the new solution. The stress fields (calculated using the virial tensor) for both dislocations match the prediction of TLE, with deviations only at the far boundary where atoms have been clamped (Fig. 1). The line energy grows logarithmically with distance; TLE’s slope is less than the new solution, and the relaxed dislocations match the TLE line energy (Fig. 2). Finally, the atoms after relaxation have a $-b\left(1-2\nu \right)/4\pi \left(1-\nu \right)\ln r/r_{0}$ displacement away from the core, as predicted by the TLE solution (c.f., information on dislocation relaxation at ref. 5). In all respects, the TLE solution correctly predicts the properties of a dislocation, while the new solution disagrees with the far-field atomistic calculation. Thus, the new solution does not correspond to an isolated dislocation at equilibrium.

**Fig. 1.**
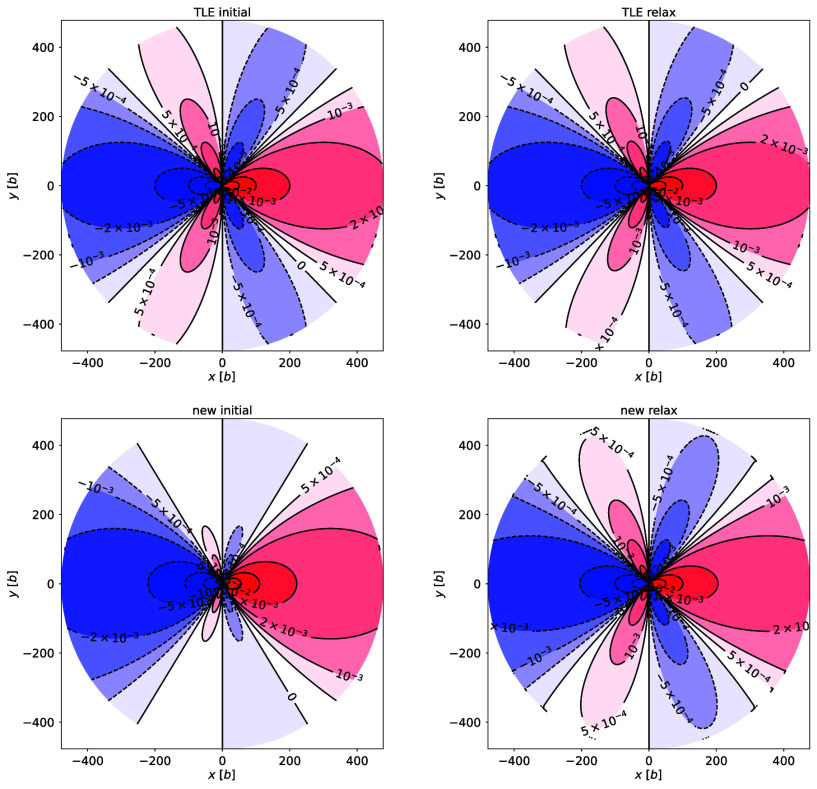
The *xy* stress field before (initial) and after (relax) relaxation for the TLE (*Top*) and new (*Bottom*) solution, in units of $\sigma_{0}=\mu b/\left(2\pi \left(1-\nu \right)\right)$. The new solution geometry begins with a different stress field than the TLE solution, but after relaxation, both dislocation geometries relax to match the TLE solution. The relaxed new solution show deviations only near the outer boundary where atoms have been clamped to the positions originally predicted by the new solution; everywhere else, the stress agrees with the TLE solution.

**Fig. 2.**
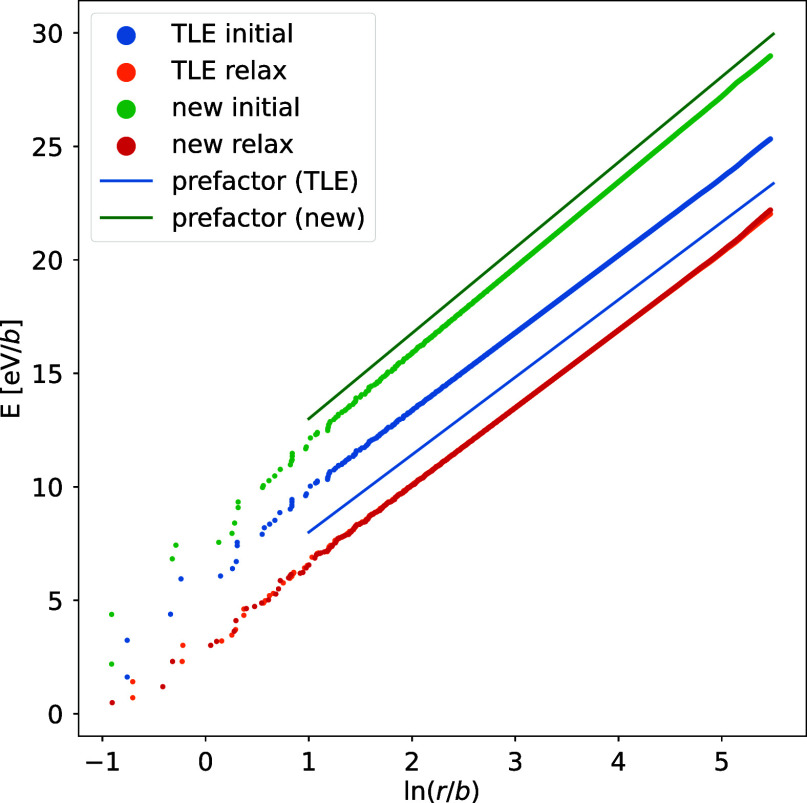
Summed atomic energy for atoms within a radius r of the dislocation before (initial) and after relaxation (relax); the slope in the far-field is predicted by elasticity theory. Relaxation corrects the core geometry for TLE lowering the energy, but leaves the long-range elastic slope the same. For the new solution, relaxation corrects the core geometry *and* the long-range elastic slope to match the TLE solution.

The data are available through the Materials Data Facility (6, 7) at DOI: 10.18126/hc1f-8y48 (5).

## References

[1] J. P. Hirth, P. M. Anderson, The revised elastic field of an edge dislocation. Proc. Nat. Acad. Sci. U.S.A. **122**, e2420808122 (2025). 10.1073/pnas.2420808122 PMC11831159 39903115

[2] J. E. Sinclair, P. C. Gehlen, R. G. Hoagland, J. P. Hirth, Flexible boundary conditions and nonlinear geometric effects in atomic dislocation modeling. J. Appl. Phys. **49**, 3890–3897 (1978).

[3] M. C. Marinica , Interatomic potentials for modelling radiation defects and dislocations in tungsten. J. Phys. CM **25**, 395502 (2013). 10.1088/0953-8984/25/39/395502 24002176

[4] A. P. Thompson , LAMMPS - a flexible simulation tool for particle-based materials modeling at the atomic, meso, and continuum scales. Comp. Phys. Comm. **271**, 108171 (2022).

[5] D. R. Trinkle, Data citation: W edge dislocation relaxation with LAMMPS, The Materials Data Facility (2025). 10.18126/hc1f-8y48.

[6] B. Blaiszik , The Materials Data Facility: Data services to advance materials science research. JOM **68**, 2045–2052 (2016).

[7] B. Blaiszik , A data ecosystem to support machine learning in materials science. MRS Commun. **9**, 1–9 (2019).

